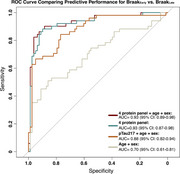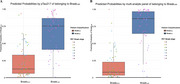# A protein panel including pTau217 outperforms pTau217 alone in identifying high tau load in amyloid positive individuals

**DOI:** 10.1002/alz70856_106338

**Published:** 2026-01-07

**Authors:** Guglielmo Di Molfetta, Wagner Scheeren Brum, Andrea Benedet, Nesrine Rahmouni, Jenna Stevenson, Ilaria Pola, Laia Montoliu‐Gaya, Kaj Blennow, Henrik Zetterberg, Pedro Rosa‐Neto, Nicholas J. Ashton

**Affiliations:** ^1^ Department of Psychiatry and Neurochemistry, Institute of Neuroscience and Physiology, The Sahlgrenska Academy, University of Gothenburg, Mölndal, Sweden; ^2^ Graduate Program in Biological Sciences: Biochemistry, Universidade Federal do Rio Grande do Sul (UFRGS), Porto Alegre, Brazil; ^3^ Institute of Neuroscienace and Physiology, University of Gothenburg, Mölndal, Västra Götaland, Sweden; ^4^ Translational Neuroimaging Laboratory, The McGill University Research Centre for Studies in Aging, Montréal, QC, Canada; ^5^ Paris Brain Institute, ICM, Pitié‐Salpêtrière Hospital, Sorbonne University, Paris, France; ^6^ Neurodegenerative Disorder Research Center, Institute on Aging and Brain Disorders, University of Science and Technology of China and First Affiliated Hospital of USTC, Heifei, China; ^7^ Department of Psychiatry and Neurochemistry, University of Gothenburg, Mölndal, Sweden; ^8^ Clinical Neurochemistry Laboratory, Sahlgrenska University Hospital, Mölndal, Västra Götaland län, Sweden; ^9^ Hong Kong Center for Neurodegenerative Diseases, Hong Kong, Science Park, China; ^10^ Department of Psychiatry and Neurochemistry, Institute of Neuroscience and Physiology, the Sahlgrenska Academy, University of Gothenburg, Molndal, Sweden; ^11^ University College London, London, United Kingdom, London, United Kingdom; ^12^ Wisconsin Alzheimer's Disease Research Center, School of Medicine and Public Health, University of Wisconsin‐Madison, Madison, WI, USA; ^13^ UK Dementia Research Institute at UCL, London, United Kingdom; ^14^ Department of Neurology and Neurosurgery, McGill University, Montréal, QC, Canada; ^15^ Banner Sun Health Research Institute, Sun City, AZ, USA; ^16^ Banner Alzheimer's Institute, Phoenix, AZ, USA; ^17^ Department of Psychiatry and Neurochemistry, Institute of Neuroscience & Physiology, the Sahlgrenska Academy at the University of Gothenburg, Mölndal, Sweden

## Abstract

**Background:**

Recent anti‐amyloid trial designs for Alzheimer's disease (AD) have aimed to identify amyloid‐β (Aβ)‐positive patients without an advanced tau pathology, as they are most likely to benefit from these therapies. Blood‐based biomarkers might reduce the need to use cerebrospinal fluid (CSF) or positron emission tomography (PET) but it is unclear whether phosphorylated tau‐217 (pTau217) alone would be effective to exclude this high‐tau group at screening. We investigated whether a blood‐based protein panel, including pTau217, could better distinguish early from late‐stage tau pathology in Aβ‐positive patients compared to pTau217 alone.

**Method:**

Aβ‐positive participants from the TRIAD cohort (*n* = 129; mean [SD] age, 70.4 [8.3] years; females [58.9%]) were classified as Braak_Late_ (Braak V‐VI: *n* = 51) or Braak_Early_ (Braak I‐IV: *n* = 78) by tau PET imaging([18F]MK6240). We employed the NULISAseq CNS Panel to quantify 120 CNS‐related proteins. A bootstrapped (1000x) LASSO regression was used to identify the most recurringly selected proteins for distinguishing Braak_Late_ from Braak_Early_. Generalized linear models (GLM) for the multi‐analyte panel and pTau217, adjusted for age and sex, were used and their performance evaluated by ROC analyses and Akaike Information Criterion (AIC) scores. GLMs were also used to estimate probability scores for each patient for belonging to Braak_Late_.

**Result:**

The bootstrapped LASSO regression retained pTau217, neuropentraxin receptor (NPTXR), vascular growth factor (VGF) and growth‐derived neurotrophic factor (GDNF) in >75% of the iterations. ROC analysis demonstrated that the multi‐analyte panel (AUC=0.93: 95% CI 0.89‐0.98) had a significantly better prediction of Braak_Late_ than pTau217 alone (AUC= 0.88; 95% CI 0.88‐0.94; *P* ^DeLong^= 0.004). The fit of the model was also assessed by comparing AIC, where the multi‐analyte panel showed a reduction in the score to detect the Braak category, suggesting a better model fit. This was further supported by an ANOVA comparison between the two models, where the multi‐analyte model was significantly better than pTau217 alone (*P* ^ANOVA^ < 0.001).

**Conclusion:**

We identified three complementary proteins (NPTXR, GDNF, VGF) to pTau217 that can improve its ability in detect later Braak stages in Aβ‐positive patients. This suggests an immunoassay‐based panel might be a cost‐effective tool to exclude participants with high tau pathology in anti‐amyloid trials designs.